# Perspectives of pregnant women on maternal health information handouts at KwaZulu-Natal sub-district

**DOI:** 10.4102/phcfm.v16i1.4158

**Published:** 2024-02-29

**Authors:** Thandi M. Dlamini, Siyabonga Dlamini

**Affiliations:** 1 Department of Public Medicine, Faculty of Health Sciences, University of KwaZulu-Natal, Durban, South Africa; 2 Cancer and Infectious Diseases Epidemiology Research Unit, College of Health Sciences, University of KwaZulu-Natal, Durban, South Africa

**Keywords:** antenatal care, perinatal outcomes, pregnant women, maternal health information handouts, patient information leaflets, patient education

## Abstract

**Background:**

Maternal health information handouts are used by midwives to facilitate health education of pregnant women during their antenatal care (ANC) period. South Africa’s Saving Mothers Report 2014 showed that delay in accessing medical help, as a patient-related avoidable factor, accounted for 27% of maternal and neonatal mortality.

**Aim:**

To ascertain the perceptions of pregnant women attending ANC in the Msunduzi sub-district in uMgungundlovu District, towards the maternal health information handouts.

**Setting:**

The study was conducted at three primary health care (PHC) clinics (two PHC and one CHC [Community Health Care]) that provided ANC in the Msunduzi sub-district KwaZulu-Natal, in 2019.

**Methods:**

Using a qualitative approach, focus group discussions (FGDs) were conducted with 10 participants from each clinic. Data were transcribed and analysed using thematic analysis.

**Results:**

The themes explored included: availability and access of handouts, usefulness, review of handouts, alternative methods available, and family involvement.

**Conclusion:**

The authors concluded that although the maternal information handouts were given to the mothers during their maternal health visits, few of them were aware of these handouts. New strategies should be employed to deliver this vital information, as suggested by mothers.

**Contribution:**

The awareness of pregnant mothers about the information handouts contributes to the positive perinatal outcomes at clinic levels.

## Introduction

Health professionals use printed materials as a primary teaching tool to give health information to patients.^1^ This is used to reinforce health information taught verbally. Patients use written health information materials for future reference.^1^ Its advantage is that readers can control the speed they use to read and comprehend information.^1^ South Africa’s Saving Mothers Report 2014 shows that delay in accessing medical help as a patient-related avoidable factor accounts for 26.9% of the adverse events; therefore, improving health education strategies is of high importance to empower pregnant women to improve perinatal outcomes.^2^ Consequently, this study aimed to explore the perspectives of pregnant women at Msunduzi sub-district towards maternal information handouts used to complement health education given by midwives to empower these women regarding their decision-making in seeking medical help.

A study done in Australia, between November 2010 and January 2011, reported that discussion with a midwife was the most useful source of information for women to fulfil their information needs during pregnancy.^3^ In addition, women who were non-English speakers were less likely to use the written information. A study of maternity centres in Wales found that only 75% of the women interviewed reported having had access to antenatal information leaflets.^4^ This study also concluded that leaflets had minimal influence in determining the choice of women using maternal services.^4^ The National Institute for Health and Care Excellence London highlighted that women raised an opinion that although there was too much written information, they still preferred the information they had received from their midwives.^5^

The study done by Singh and associates in 2016 found a substantial difference in the influence of health education using integrated leaflet distribution models, demonstrations and the use of audio-visual devices, compared to isolated leaflet distribution.^6^ A study conducted in the rural India by Gupta in 2016 concluded that students preferred audio-visual media and PowerPoint presentations, rather than leaflets for their lectures because pamphlets were brief and get easily misplaced; but, the other two methods were more interesting because of the integration of pictures, animations, among others.^7^ Experiences of pregnant women are different from those of students; however, some lessons can be learned from this article, especially regarding the use of visual materials.

A study conducted in both rural and urban areas of Gambia showed that information, education, and communication during antenatal care (ANC) in the largest health division were inadequate.^8^ However, it is not clear if this study included written patient information; but, the results showed that pregnant women were not fully equipped to make appropriate decisions during critical periods. This contributed to the persistence of high maternal mortality ratios in the country.^8^

A point which one may make from the above-mentioned several studies is that in order for the health information handouts to be useful, they need to be used together with other health educational methods. In low- and middle-income countries (LMICs), health education programmes usually fail for several reasons. One study categorised four interrelating groups of explanations of why the health education plans tended to fail.^9^ ‘Failures in planning process to apply epidemiological and behavioural sciences to the selection of appropriate objectives’, failure in communication in terms of reaching the desired audience, and failure in promoting adequate understanding as well as acceptance of what was communicated.^9^

In South Africa, maternal health information handouts are used as an additional source of information given by midwives to pregnant women. As stated in the Guidelines for Maternity Care in South Africa, ‘Certain essential information must be provided to all pregnant women, verbally and (where possible) in the form of written or illustrated cards or pamphlets’.^10^ The information given to pregnant mothers varies from caution about danger signs to the instruction regarding what the women are expected to do should they experience any of these danger signs for instant reporting to the nearest health facility immediately carrying their maternity case records.^10^ A study conducted in Tshwane argued that verbal midwife education should be supplemented by utilising health promotion and counselling leaflets.^11^ The use of symbols and pictograms were found being helpful in assisting patients to easily find and access information.^12^ Studies have shown that written information accompanied by pictures are the preferred format for the presentation of medicine instructions in South Africa.^12^ This may add to the easy reading and understanding of the instructions.

The basic objective of ANC is to ensure positive perinatal outcomes through screening assessment, treatment, and giving information to pregnant mothers.^13^ Health education is described by World Health Organization (WHO) 2016 ANC model,^13^ ‘as the model that is aiming at providing pregnant women with respectful, individualized, person centred care’. Early ANC attendance provide midwives an opportunity to detect and treat complications timely.^14^ The late booking (first antenatal visit after 20 weeks gestational period) still remains a huge problem among pregnant women in South Africa despite the provision of free ANC services.^15^ Maternity case records are a standardised document used by ANC centres in South Africa, which is received by all pregnant women that present themselves to a health care facility, public or private, for ANC.^10^ It is completed by midwives and health care professionals in each and every visit to ensure continuity of care within levels of care. The maternity case record is kept by the mother till delivery. Maternity case records are also used to attach maternal health information handouts which the current study is exploring.

There are quite a number of models that have been developed to explain human behaviour. These are health literacy model, the rational model, the health belief model, the extended parallel process model, the transtheoretical model of change, and the activated health education model.^16^ The health literacy model was used in this study to explore how pregnant women used written maternal health information handouts to make decisions about seeking medical assistance during their pregnancy. The health literacy model is the model of choice in the current study since it is the pathway model which describes how health literacy enables people who are being supported by others, to seek, engage with, and act on health information to manage their health and become actively involved in health.^16^ The model also gives a description of the outcome of health education and communication activities.^16^ Thus in the current study, the health education using maternal information handouts is meant to support and assist pregnant women in seeking medical help timeously to prevent adverse outcomes. The health literacy model uses the health outcome model which highlights health literacy as the key outcome from health education.^16^

**FIGURE 1:**
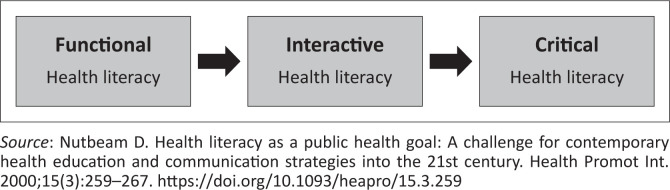
Health literacy model.

Health literacy is defined by the WHO as ‘more than being able to read a pamphlet and successfully honouring one’s appointments, as it is also about improving access to health information and the capacity to use it effectively’.^17^ Health education is meant to improve health literacy. In the current study, decisions about when pregnant women seek medical help were explored. The health literacy model consists of three aspects (see Figure 1^16^): (1) The functional health literacy, which educates patients on the health risk to reach the educational goal. In this study, the knowledge of pregnant women on the related health risks was explored, as it might have an impact on their decision-making. (2) The interactive health literacy, which focusses on developing individual skills in a supportive environment, thus improving independent personal decision-making and good behavioural outcomes. This study explored the perceived support the pregnant women received during ANC visits to empower them with the necessary skills to make independent decisions leading to positive pregnancy outcomes. (3) The critical health literacy, which aims to improve individual decision-making on social determinants of health.^16^ This is directed towards individuals and the community. In the current study, social determinants of health perceived by the pregnant women as impacting on them delaying seeking medical help were explored.

## Research methods and design

### Study design

A qualitative phenomenological study was conducted by the researcher to gain an in-depth understanding of the pregnant women’s perception towards maternal health information handouts.

### Research setting

**FIGURE 2:**
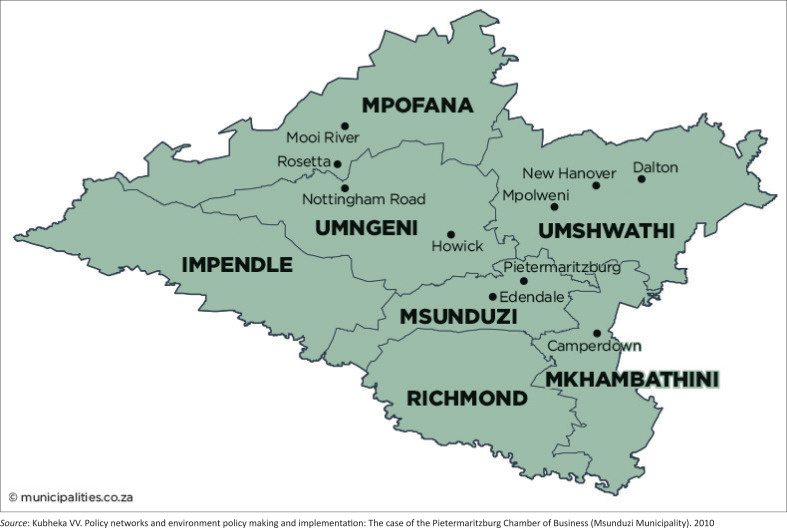
Msunduzi local municipality map.

Primary Health Care (PHC) clinics that provide ANC services at Msunduzi sub-district were used. Three PHC sites were chosen, namely: (1) Imbalenhle Community Health Care (CHC) Centre with a Midwifery Obstetric Unit, (2) Taylors Clinic, which is a 24 h PHC clinic with a labour facility, and (3) Caluza Clinic, which is a 24-h PHC clinic. All three PHC facilities are in the Msunduzi sub-district located within uMgungundlovu District, KwaZulu-Natal (KZN). Msunduzi sub-district is among the seven municipalities of the district (Figure 2^18^). It includes the city of Pietermaritzburg, which is the capital of the province and the main economic hub of the district.^18^

According to Stats SA Census 2011, the municipality has a population of over 616 730 and 163 993 households of which 45.2% are headed by females. It has 73.7% of formal dwelling, of which 47.9% have access to piped water inside the house.

### Population and sampling

Pregnant women from the age of 18 years upwards attending ANC services at the three identified PHC facilities in Msunduzi sub-district were included in the study. Ten participants per facility were selected. Pregnant women coming for subsequent visits were chosen to participate as they had been exposed to the handouts and had used them. Pregnant women visiting the identified PHC facilities for the first time were not selected, as they had not been exposed to the handouts as yet. Women who were not pregnant were not included. All females younger than 18 years and all men were excluded.

### Data collection and tools

The researcher conducted focus group discussions (FGDs) to collect data – 10 participants in a focus group per day in each facility. Pregnant women from the age of 18 years upwards attending ANC services at the three identified PHC facilities in Msunduzi sub-district were included in the study. Ten participants per facility were selected by taking the first 10 meeting the criteria, provided they were willing to participate in the study. The study was done over three consecutive working days in the mornings from 18 March 2019 to 20 March 2019, and the discussions lasted about 45 to 60 min. A private room was organised by the nursing staff at the respective facility where each FGD was conducted.

A discussion guide was developed by the researcher beforehand to guide the discussions.^19^ Furthermore, the discussion guide was developed by identifying key issues from the literature and discussions with the study supervisor. The study tool was also assessed by an experienced qualitative researcher in the field of public health. The purpose of the study was explained to the participants in their language of preference. Informed consent was obtained from the participants after the information sheet written in isiZulu was handed over to them. Participants gave a verbal consent on the voice recordings; they were then allowed to deliberate on the phenomenon, voice their subjective and objective views on the issues discussed, and make recommendations on the written maternal health information handout. The FGD was carried out in IsiZulu since that was the language the participants were comfortable with. At the beginning of the discussion, the researcher introduced herself where she works and the university she is coming from. The topic was then fully introduced to the participants. The issues of confidentiality and consent forms were discussed. Throughout the discussion, probing and questions to clarify issues were implemented. The participants were informed that while the discussion is audio recorded some notes will be taken while the discussion is going on to make sure that important facts were not missed. At the end of the discussion, refreshments in the form of fruits and juice were given to participants since they were delayed by the discussions.

### Data analysis

A thematic framework data analysis method was used to analyse the data.^20^ Although Anderson advocates for doing this process with hard copies, we conducted it electronically using NVivo 12 software (LUMIVERO, Denver, Colorado USA). The thematic analysis approach is used to explore the meaning that participants attached to their lived experiences. The first step was to group similar ideas together by identifying and marking areas with distinct units of meaning. These units of meaning were assigned codes. Codes that were similar and went together were categorised into themes using keywords or phrases from the text. This process was done iteratively with all the transcripts, where grouping and regrouping of units of meaning were done until sense could be made of these emergent themes and sub-themes. Thereafter, the sub-themes were collapsed into themes to have fewer sensible categories, and were reviewed to ensure that meaning was not lost in the process. This process was repeated to ensure rigour and the retention of meaning.

### Trustworthiness

A thick descriptive narrative was developed from the collected data so that judgements about the degree of fit or similarity can be made. In this study, the researcher encouraged a true reflection of the participants’ views by developing a trusting relationship and establishing rapport with the participants from recruitment to the end of the data collection process. The trusting relationship was achieved by using a friendly tone while greeting the participants.

Dependability was addressed by reporting the study process in detail, including the research design and its implementation (operational details such as recruitment and data gathering) and reflective project appraisal. Triangulation was ensured by allowing the co-author to read the transcripts and check the interpretation of data.

The researcher’s reflexivity was accounted for by keeping a journal to understand how her experiences affected the research process. Confirmability was reached by making raw data accessible and conducting member checking with participants where necessary. The study’s accuracy was ensured by creating and maintaining an audit trail. This captured the entire study process and ensured that the data was securely stored and easily accessible, should the need arise. A report on how the data were transcribed is kept and may be provided if needed.

The researcher was born in Durban and grew up in Ixopo area in a place called Lufafa. She did her basic education in Ixopo and moved to Pietermaritzburg to complete secondary education at Kwa Pata High school where she got her Matric in 1996. She then moved to Johannesburg to pursue her career at the University of the Witwatersrand where she graduated with a Bachelor of Nursing in 2001. She started working as a midwife in 2002 at Mahatma Gandhi Memorial Hospital. In 2006, she completed a post-graduation Diploma in Advanced Midwifery and Neonatal Nursing Science, and has been practising as an advanced midwife since then. In 2010, she completed an Advanced University Diploma in Health Science Education through the North-West University. At the time when the research was conducted, she was occupying a position of Assistant Manager Nursing for Obstetrics and Gynaecology at Harry Gwala Regional Hospital (formerly known as Edendale Hospital). The researcher became interested in this topic because she needed to get an in-depth understanding of the perspectives of pregnant women about whether the handouts that are given to them during first antenatal visit were helpful or not, and to find out alternative ways to enhance them to use these handouts to their full capacity. Added to the relationship of the researcher and the participants was that the researcher was from the hospital where these patients are referred to for delivery if they face complications, and that built the trust between them. A journal was kept to deal with issues of researcher’s subjectivity.

### Ethical considerations

The study obtained ethics approval from the Biomedical Research Ethics Committee of the University of KwaZulu-Natal (BE656/18) and the KwaZulu-Natal Department of Health (NHRD Ref: KZN_201811_030). Permission to conduct the study was obtained from the participating health facilities. The participants gave a written informed consent before participating in the study.

## Results

Thirty pregnant women of the age of 18 years and above attending ANC services at the three identified PHC facilities in Msunduzi sub-district were included in the study. The analysis process yielded the following themes: availability and access, usefulness, review of handouts, alternative methods available, and family involvement.

### Availability and accessibility of handouts

In the Guidelines for Maternity Care in South Africa, it is stipulated that certain important information should be provided to all pregnant women verbally and in the form of written or illustrative cards or pamphlets.^10^ All the public health sector facilities providing ANC attach pamphlets with written maternal health information on maternal case records. These pamphlets are meant to assist pregnant women in deciding when and where to seek medical assistance. The current study demonstrates that most of the participants have seen these handouts attached to their maternity case records. In the words of one of the participants:

‘It has information on the danger signs and what you should do if you are faced with that situation.’ (Participant A, 35 years, Street vendor)

Out of 30 participants, 8 said that they were not given the handouts. As stated by a participant:

‘I was not given the handouts because I do check and peruse my maternity case record.’ (Participant B, 25 years Domestic worker)

From Taylors Halt Clinic, it emerged that some of the participants were given photocopied maternity case records without the handouts attached to them. Participant N put it as follows:

‘We do not have a complete set of handouts since we were given photocopied maternity case records.’ (Participant N, 35 years Housewife)

### Usefulness

The handouts are attached to supplement health education given to pregnant women in order to improve perinatal outcomes. This study found that the handouts appeared to improve pregnant women’s knowledge and provided a guide on when to report to the clinic should problems be encountered. For instance, Participant C said:

‘If these handouts were not there, we would not know … because sometimes at the clinic you are not taught about some other things but taught on other things. Therefore, the handouts help you when you are at home. And, if you are experiencing one of those danger signs, you would know you have to rush to the clinic.’ (Participant C, 30 years, Hairdresser)

### Review of handouts

The participants were asked to share their views on the handouts. The majority felt that the midwives should ask them regarding the handouts on their ANC visit to ascertain if they were using it and to find out if the pregnant women were aware of such handouts in their maternity records. In the words of a participant:

‘The nurses should check if we read it because some of us at home just put the handouts in the bags and leave it there and never read it.’ (Participant D, 35 years, Unemployed)

It was pointed out that the way these handouts are attached to their maternity case records was not user-friendly. Participant A argued that:

‘… these handouts are so important, in my opinion, but they are attached right at the back of the maternity case record where we do not easily see them. But, if they can be attached at the front of the maternity case record we will be able to access them easily when at home other than perusing or going through the whole file.’ (Participant A, 35 years, Street vendor)

### Family member’s involvement

The participants were asked regarding the family members’ involvement and the use of the handouts in assisting them when seeking medical help. There were different opinions on this. Some felt it was a good idea and stated that their family members knew about the handouts and read them, but some felt that it was unnecessary for family members to read the handouts because it was theirs and applied to them. For example, Participant E said:

‘My maternity case record is not hidden, and my family members are even interested in knowing about the foetal kick count chart they want to know what is happening. As I am here today, they will request feedback on what is going on if there are any changes, bad or good.’ (Participant E, 40 years, Domestic worker)

Others were worried about the confidentiality of their illness and/or condition being disclosed to their family members.

‘It is okay for her to hear about that from me, but not that I will give her my maternity case record. It is none of her business. Explaining it to her is enough.’ (Participant F, 23 years, Student)

Participant G added:

‘Some people at home will read your secrets about your illness, and that’s why someone will hide her maternity case record.’ (Participant G, 29 years, Cleaner)

### Recommendations on health education

The participants suggested the use of short text messages, as per the Mom Connect Project. This programme is currently implemented by Department of Health in South Africa to send messages to pregnant women’s cell phones, nationally, to remind them of important things:

‘Yes, there is this programme called Mom Connect which in my opinion has played a big role in giving us messages even if we are not at home and my maternity case record is not with me. If maybe these information handouts can be summarised and sent through our cell phones as messages.’ (Participant A, 35 years, Street vendor)

‘What I can suggest is what if they send it to us on our cell phone through short text messages same as Mom Connect project because we do not have time to read, we just read the return date.’ (Participant M, 22 years, Student)

Others felt that being taught by midwives, whether individually or in groups, regarding the pamphlets could improve their awareness and the use of the handouts:

‘Maybe in the morning while we are still waiting if one nurse can come and take us through, teaching us about what is on these handouts same way as they do with Mom Connect in the morning. I do not think it can take even more than five minutes to remind us.’ (Participant M, 22 years, Student)

‘It is known that as black people if you want to hide something from us one must put it inside a book because reading is not our thing.’ (Participant H, 29 years, Unemployed)

## Discussion

The Maternity Care Guidelines of South Africa recommend that written information must be attached to the patient maternity case records as supplementation to verbal patient education, and these need to be attached to the maternity case record on the first visit.^10^ However, the current study found that the information handouts were available, but that they were not always given to pregnant women on their first visit. About 30% of the pregnant women in this study did not receive the handouts on their first visit, while others were given photocopied maternity case records and only received the handouts on subsequent visits. About 16% of mothers said they received the handouts during the FGD.

Accessing the handout on the maternity case record was a problem for some women, suggesting that nurses should attach these handouts on the front of the maternity case record, not at the back, so that it is easy to access them at home. Another suggestion was that the nurses should ask about these handouts when mothers come for subsequent visits to find out if mothers do read them at home.

The South Africa Saving Mothers Report 2014 commented that among avoidable patient-related factors to poor perinatal outcomes, delaying access to medical help accounted for 27% of avoidable patient-related factors leading to poor perinatal outcomes.^2^ However, in the current study the majority of women stated that information handouts were important in providing them with useful information and directly assisted them to understand their health needs and when to seek medical help.

In this study, even though the majority of the women were aware of the handouts in their maternity records, they still delayed seeking medical help because of different reasons. Some mentioned that they did not read the handouts, while others felt it was still the primary role of the midwife to teach them. These findings are similar to an earlier study done in South Africa, where pregnant women were expected to learn about their health concerns and needs from health care workers.^11^ In a study done in the United Kingdom, it emerged that access to health records by patients had a small impact on the patients’ health behaviour, and instead reinforced trust and confidence in doctors, and helped patients to feel like partners in health care.^21^

Mothers suggested that big posters with the same information in the handouts be made for mothers to read on their own while in the waiting area. The findings further reveal that pregnant women require varied information sources; for example, the use of SMS similar to the Mom Connect programme for them to comprehend ANC information.

Various studies both in the developed and developing world allude to this. For example, a study in Canada revealed that the education of pregnant women was of significance for pregnant women to make informed decisions regarding their health.^22^ Furthermore, studies revealed that information can be varied, including video, audio, computer and print, as long as the information is based on evidence.^23,24^ The African continent is challenged when it comes to the use of short messages as a mode of communication. People change their cell phone numbers often because of different reasons, such as stolen cell phones, cheap and damaged cell phones, and migration from one network to another because of poor network coverage in that area.

Women, families and communities need to be empowered in order for them to contribute positively towards perinatal health. The current study showed that families do play an important role when it comes to pregnant women, which helps them make informed decisions.

The need for raising awareness among pregnant women about the handout was highlighted for the women to be aware of what is in the maternity file so that they can read it when they get home. Others mentioned that, as black people, they normally do not read written material, and that midwives should do a 5 min quick check to see if they are reading these handout at home and ask questions if need be.

### Limitations of the study

There was limited literature on the written maternal health information handouts. Some of the demographic data (i.e., educational status of the participants) were not collected, which could have provided more context to the study. After the completion of data collection for this study, the South African National Department of Health developed new maternity case records in 2019, as an effort to enhance the use of these handouts by pregnant women. Therefore, research on this new maternity case record is needed. The researcher has a background on using the handout herself while in the practice which might impact on the interpretation of the result, but this was addressed through the researcher keeping a reflexive journal. Because of the limited resources for this study, the number of focus groups was predetermined. Therefore, we cannot claim that data saturation was reached. However, this study provides valuable insights into the experiences and the behaviour of pregnant women regarding these handouts. A similar study can be conducted in the future to explore these findings in greater depth. The findings of this study may be transferrable to resource-limited, rural settings.

### Recommendations for practice

- The midwives should help raise the awareness of the attached additional information handouts by informing women where to locate the handouts in their maternity case records, as well as informing them how to use the handouts and when it will assist them; for example, when they experience medical problems.
- In 2019, South Africa developed a new maternity case record that has most of the handouts attached to it. However, these handouts are used by health care workers for patient care. This information could be used to empower pregnant women about their pregnancy.
- Use of short messages to educate on pregnancy, as this is already used by Mom Connect, was recommended by other women in the current study.
- The midwives should teach mothers how to read and use the attached handouts at home. This includes having a separate pack for family members at home to assist the pregnant women in making informed decisions on where and when to seek medical assistance, should the need arise.

### Recommendations for future research

This study explored the perspectives of pregnant women towards maternal health information handouts at Msunduzi sub-district. However, sampling was done in only three PHC clinics, and a more representative selection of participants across the district would improve the understanding of the phenomenon under study. The South African National Department of Health had developed the new maternity case records in 2019 as an effort to enhance the use of these handouts by pregnant women. Therefore, research on this new maternity case record is needed.

## Conclusion

Pregnant women at Msunduzi sub-district are aware of the maternal information handouts attached to their maternity records. However, barriers to using them exist, such as the position where these handouts are attached at the back of the maternity case records. Another aspect was that of pregnant women not having a penchant for reading, and that it would therefore be better if the midwives conducted lessons regarding the use of such handouts. Also, women only realise late in their pregnancy that they are pregnant. Finally, some women prefer reading text messages to something written on paper. This technology is already being used in other health programmes in South Africa, and could be applied to these maternal information handouts as well.
